# Supplementary figures and images for: The Sphingosine-1-phosphate pathway is differentially activated in human gestational tissues
Source: bioRxiv. 2025 Jun 5:2025.06.02.657487. Preprint. [Version 1] doi: 10.1101/2025.06.02.657487 (PMC12157600; doi:10.1101/2025.06.02.657487)

A

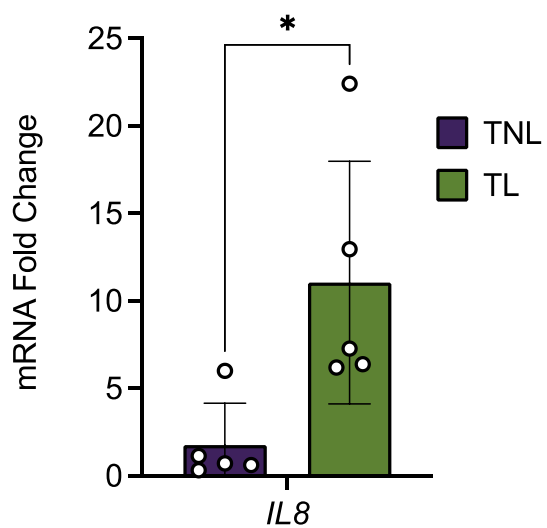

Supplement: Supplement 2 — Supplemental Fig 1. An inflammatory marker of parturition in the myometrium of term non-labor (TNL) and term-labor (TL) human tissue. mRNA expression of IL-8 in the myometrium of TNL (n=5) and TL (n=5) patients was analyzed by quantitative real-time PCR (RT-PCR). Presented are means ± SD. Statistical significance was determined by using multiple unpaired t-test with Welch correction (*P < 0.05). [file media-2.pdf]
